# Prevalence of Internet Addiction and Its Associated Risk Factors Among Medical Students in Sudan: A Cross-Sectional Study

**DOI:** 10.7759/cureus.53543

**Published:** 2024-02-04

**Authors:** Khalid O Mohamed, Sozan M Soumit, Aziza A Elseed, Wamda A Allam, Amro M Soomit, Humeda S Humeda

**Affiliations:** 1 Internal Medicine, Faculty of Medicine, University of Bahri, Khartoum, SDN; 2 Internal Medicine, Faculty of Medicine, Sudan International University, Khartoum, SDN; 3 Medical School, Faculty of Medicine, National University, Khartoum, SDN; 4 Pharmacy, Faculty of Pharmacy, Omdurman Islamic University, Omdurman, SDN; 5 Urology, The Arab Board of Health Specializations, Khartoum, SDN; 6 Physiology, Faculty of Medicine, Alzaiem Al-azhari University, Khartoum, SDN

**Keywords:** prevalence, medical student, psychological distress, sudan, internet addiction

## Abstract

Background

Internet addiction has been studied extensively worldwide and shown to have adverse social, psychological, and functional consequences. It has become a global health issue, particularly among young adults. Unfortunately, few studies have been conducted among medical students in Sudan. This study aimed to measure the prevalence of internet addiction and determine its associated risk factors among medical students in Sudan.

Methods

This cross-sectional study was conducted among medical students using a systematic sampling technique. An online structured questionnaire was used, which included sociodemographic data and the Internet Addiction Test (IAT) to assess the presence and severity of internet addiction, as well as the Kessler Questionnaire to assess psychological distress.

Results

Among the 307 medical students who participated in this study, 63.8% (n=196) were female. The majority 78.5% (n=242) were aged 18-22 years, and the prevalence of internet addiction was 75.5% (n=232). The addiction levels were as follows: mild, 39.7% (n=122); moderate, 33.2% (n=102); and severe, 2.6% (n=8). Multivariate regression analysis revealed that being a female medical student was associated with a 1.9 times higher risk of internet addiction (adjusted odds ratio (AOR) = 1.87, p = 0.033). Psychological distress was associated with a 6.368-fold higher risk of internet addiction compared to non-distressed students (AOR = 6.368, p < 0.001). Additionally, students in the clinical years had a lower risk of internet addiction, specifically in the fourth and fifth years (AOR = 0.325, p = 0.010; AOR = 0.398, p = 0.043, respectively).

Conclusions

This study found a high prevalence of internet addiction among medical students at the National University, Khartoum, Sudan, which was strongly associated with mental distress. Effective awareness programs, potentially initiated by educational authorities, are required to educate students on limiting excessive internet usage and addressing associated risk factors. Future research should include longitudinal and multi-university studies to obtain more generalizable results and to investigate the relationship between internet addiction and mental distress more robustly.

## Introduction

The internet has become essential to daily life due to its rapid technological advancement over the past 20 years. When used appropriately, it offers cutting-edge communication and access to vast knowledge, making life easier [1].

Technology has become ubiquitous in almost every facet of modern life, and the internet is no exception to its widespread presence and use among all generations. The application of digital technology has numerous advantages, dramatically transforming the current generation's lifestyle and making life easier and more enriched in many ways. However, this technological advancement comes with the high costs and risks associated with its overuse. The consequences of inappropriate internet use can be seen in all users; however, adolescents are particularly vulnerable. Overuse of the internet can quickly turn into abuse, interfering with an individual's ability to function and perform ordinary tasks. Certain aspects of excessive internet use have been increasingly recognized as harmful. Specifically, in the context of internet addiction, individuals may develop obsessive and addictive behaviors [2].

The available data indicates that the internet penetration rate in Sudan was 30.9% in January 2022, with 14.03 million internet users reported. This figure represents a 2.4% increase between 2021 and 2022 [3].

In Sudan, a study conducted in 2016 among high school students concluded that internet addiction is a concerning issue that requires interventions, especially from families, to minimize its impact [4].

Although internet addiction is not classified as a mental disorder according to the Diagnostic and Statistical Manual of Mental Disorders, 5th edition (DSM-5), it is widely considered a concerning disorder due to neural abnormalities and cognitive dysfunctions (e.g., impaired working memory) associated with it, which are similar to those seen in substance and behavioral addiction [5].

Furthermore, research has indicated that internet addiction is particularly prevalent among medical students. A study conducted at Tanta University in Egypt found that 51.7% of medical students were severely addicted, while only 11.3% of non-medical students exhibited severe addiction [6].

The prevalence of internet addiction among medical students is a topic of growing concern. A meta-analysis of 3,651 medical students revealed that the global pooled prevalence of internet addiction is 30.1% [7]. However, the prevalence rate of internet addiction varies widely across different countries and studies. For example, a study conducted on medical students in Egypt found that 68.3% of students were internet addicted [8], while studies among medical students in India reported a prevalence rate of 58.87% [9], in Tanzania, 31%[10] in Iran, 22.8% [11], in Korea, 11.5% [1], and in Saudi Arabia, 12.4% [12].

Medical students are particularly susceptible to addiction as they are required to use the internet for prolonged periods due to the significant demands of their academic requirements, such as conducting research, completing homework, and attending online lectures. Studies have shown that medical students who spend too much time on the internet are significantly addicted to the internet [13].

Several risk factors for internet addiction have been identified. The most well-known risk factor is gender, with males having a five-fold higher risk of addiction than females [14].

This may be due to the fact that female college students may receive better parental supervision than male students. In addition, individuals who are socially inactive or dissatisfied with offline contacts are more likely to use the internet, and individuals with disabilities are more likely to be addicted to the internet [15].

Previous research has identified associations between internet addiction and both poor social skills and social anxiety [16]. Additionally, internet addiction has been found to have a significant negative impact on life satisfaction and engagement [16], as well as academic performance [17].

Previous studies have demonstrated a significant association between internet addiction and psychiatric symptoms, including depression, anxiety, and stress. Medical students who have internet addiction showed a higher prevalence of depression and anxiety compared to non-addicted students [18,19].

It has been documented that internet addiction has a significant association with mental health and may cause psychological distress, especially among medical students [15,20].

However, the extent of internet addiction among medical students in Sudan has not been well documented. A more accurate understanding of this issue could help guide interventions aimed at preventing and managing internet addiction.

The purpose of this study was to assess the prevalence of internet addiction among medical students at the National University in Khartoum, Sudan, and to explore its relationship with various sociodemographic characteristics and psychological issues. This article was previously posted as a preprint on the Research Square preprint server on January 19, 2024.

## Materials and methods

Study design and setting

This cross-sectional study was conducted among National University undergraduate medical students in Khartoum, Sudan, between October and December 2022. A list of students from all academic years was accessed, and respondents were selected using the systematic sampling method. Only the selected students were sent a link to a Google Form containing the questionnaire. Each respondent was asked for consent before answering the questionnaire, and only those who gave consent were allowed to proceed to the next page.

Sample size

Cochran's formula was used to calculate sample size: Z² * p̂(1-p̂)/E², where n = sample size, Z = Z Value, p̂ = population proportion, and E = margin of error. The minimum final sample size was estimated to be 305 medical students.

Sampling technique

Participants were selected using a systematic random sampling technique. We obtained the list of all students and used a random number generator to generate a starting point between one and five. We then selected every fifth student to participate in the questionnaire.

Data collection tool

Data was collected through a self-administered structured questionnaire consisting of three parts. The first part included socio-demographic factors (age, gender, marital status, residence, and academic year). The second part focused on assessing internet addiction, utilizing the Internet Addiction Test (IAT) questionnaire. This questionnaire is a validated and reliable assessment tool that is widely used to diagnose internet addiction, ranging between mild, moderate, and severe levels [17]. The questionnaire comprises a 20-item scale on a five-point Likert scale, where 0 = not applicable, 1 = rarely, 2 = occasionally, 3 = frequently, 4 = often, and 5 = always. The severity of the illness according to the IAT is as follows: 0-30 points is "no addiction," 31-49 points is "mild addiction," 50-79 points is "moderate addiction," and 80-100 points is "severe addiction"; scores above 30 were considered indicative of addiction [21]. The third part assessed psychological stress using the Kessler Psychological Distress Scale (K10), a 10-item questionnaire that evaluates depression and anxiety symptoms experienced in the past four weeks [22].

Ethical consideration

Ethical approval for this study (Research Ethical Committee No: 20) was provided by the Institutional Review Board of the Faculty of Medicine, International University of Africa, Khartoum, Sudan, on April 17, 2022. Permission to conduct the study was obtained from the Dean of the Faculty of Medicine, National University. Informed written consent was obtained from each participant at the beginning of the questionnaire. Participants were informed that their participation in the study was voluntary and that their confidentiality would be protected. 

Data management

Data were analyzed using IBM SPSS Statistics for Windows, Version 26, (Released 2019; IBM Corp., Armonk, New York, United States). Continuous variables were described as mean ± standard deviation, while categorical variables were described by frequency and percentage. A multivariate regression analysis was used to determine the risk factors of internet addiction. A p-value of less than 0.05 was considered statistically significant.

## Results

A total of 307 medical students participated in this study. Most of them were female, at 63.8% (n=196). The majority, 78.5% (n=241), were 18-22 years old. Additionally, 97.4% (n=299) were single. About 72.3% (n=222) lived in the Khartoum locality. Approximately 44% (n=135) lived with their first-degree relatives. A total of 18.9% (n=58) were first-year students, 19.9% (n=61) were second-year students, 19.2% (n=59) were third-year students, 22.1% (n=68) were fourth-year students, and 19.9% (n=61) were fifth-year students (Table 1).

**Table 1:** Socio-demographic characteristics of study participants, N=307.

Variable	Category		n	%
Gender	Male		111	36.2%
	Female		196	63.8%
Age group	Less than 18		26	8.5%
	18-22		241	78.5%
	23-25		36	11.7%
	Above 25		4	1.3%
Marital status	Single		299	97.4%
	Married		8	2.6%
Residency	Khartoum		222	72.3%
	Bahri		45	14.7%
	Omdurman		40	13.0%
Academic years	First year		58	18.9%
	Second year		61	19.9%
	Third year		59	19.2%
	Fourth year		68	22.1%
	Fifth year		61	19.9%
Living situation	Live with friends		97	31.6%
	Live with first-degree relatives		135	44.0%
	Live in dormitory		20	6.5%
	Live with second-degree relatives		55	17.9%

Prevalence of internet addiction among medical students

The mean IAT score was 43.9 ± 18.9, and the range was from 4 to 100. According to the IAT scale, the prevalence of internet addiction was 75.5% (n=232). The level of addiction was as follows: 39.7% (n=122) were mild addicts, 33.2% (n=102) were moderate addicts, and 2.6% (n=8) were severe addicts, as shown in Table 2.

**Table 2:** Distribution of internet addiction and its level among the participants, N=307.

Internet addiction	n	%
No addiction	75	24.4%
Mild addiction	122	39.7%
Moderate addiction	102	33.2%
Severe addiction	8	2.6%

Risk factors for internet addiction among medical students

A multivariate regression analysis was conducted to predict the occurrence of internet addiction, considering various risk factors. The results revealed a significant association between internet addiction and being a female medical student (adjusted odds ratio (AOR) = 1.87, p = 0.033). Psychological distress was associated with a 6.368-fold higher risk of internet addiction compared to non-distressed students (AOR = 6.368, p < 0.001). Additionally, students in the clinical years, specifically in the fourth and fifth years, had a lower odds ratio for internet addiction than other academic years (AOR = 0.325, p = 0.010; AOR = 0.398, p = 0.043, respectively) (Table 3).

**Table 3:** Multivariate analysis of internet addiction risk factors.

Predictor variable	Category	p-value	Adjusted odds ratio (AOR)	95% CI for OR
Gender	Male	1.000 (Reference)	-	-
	Female	0.033	1.874	[1.052, 3.340]
Academic year	First year	1.000 (Reference)	-	-
	Second year	0.148	0.542	[0.236, 1,242]
	Third year	0.102	0.500	[0.218, 1.147]
	Fourth year	0.010	0.325	[0.139, 0.762]
	Fifth year	0.043	0.398	[0.163, 0.972]
Psychological distress	Not distressed	1.000 (Reference)	-	-
	Distressed	0.000	6.368	[2.704, 14.997]
Age group	Less than 18 years	1.000 (Reference)	-	-
	18 to 22 years	0.718	0.683	[0.086, 5.413]
	More than 22 years	0.894	0.864	[0.099, 7.531]
Marital status	Single	1.000 (Reference)	-	-
	Married	0.478	0.574	[0.124, 2.659]
Living situation	Live with friends	1.000 (Reference)	-	-
	Live with a first-degree relative	0.648	0.868	[0.474, 1.592]
	Live in dormitory	0.103	2.450	[0.835, 7.193]
	Live with second-degree relatives	0.739	| 0.880	[0.414, 1.869]

Psychological distress levels among medical students

The mean score of the Kessler scale was 25.7 ± 7.9; the range was 10 to 47. According to the score, 22.8% (n=70) of the students were well, 22.1% (n=68) had a mild mental disorder, 18.9% (n=58) had a moderate mental disorder, and 36.2% (n=111) had a severe mental disorder (Table 4).

**Table 4:** Distribution of psychosocial distress among the participants, N=307.

Level of distress	n	%
Well	70	22.8%
Mild mental disorder	68	22.1%
Moderate mental disorder	58	18.9%
Severe mental disorder	111	36.2%

A Pearson’s correlation analysis was conducted, which indicated a strong, positive, and significant correlation between psychological distress and internet addiction among the students (r = 0.594, p = 0.00) (Table 5).

**Table 5:** Correlation between the psychological distress score and Internet Addiction Test score, N=307.

Independent variable	R-value	p-value	Direction of relationship
Psychological distress	0.594	0.00	Positive

## Discussion

Over the past few decades, technology has advanced dramatically. With the widespread availability of the internet among young people, it is now easy for them to spend extended hours online. Unfortunately, this can adversely affect their ability to function in various aspects of their lives. A systematic review and meta-analysis conducted by Noroozi et al. revealed that internet addiction has severe implications for psychological, physical, and overall quality of life [5]. Additionally, studies have indicated that medical students are particularly susceptible to internet addiction, with a prevalence rate five times higher than that of the general population [7]. The present study examines the level of internet addiction among medical students in Sudan and explores its associated risk factors.

According to our study, we found that the prevalence of internet addiction among medical students was 75.5%, with a severe addiction rate of 2.6%. When comparing our results to similar studies conducted in other countries using the same measurement tool, we observed that our prevalence rate was higher than that reported among Ethiopian undergraduate students, which was 53.6%, with a severe addiction rate of 2.1% [23]. Similarly, our prevalence rate was more significant than that among Iraqi medical students, where it was 71.8% with no severe addiction reported [18]. Additionally, our results exceeded those of a recent study conducted in North Iran, which found an addiction rate of 22.8% and a slightly higher severe addiction rate of 3.4%, which aligns more closely with our findings for severe addiction [11]. Furthermore, a study conducted by Chaudhari et al. among medical students in India revealed a prevalence rate of 58.87%, with no students classified as severely addicted [9]. Moreover, a meta-analysis of African high school and university students reported an overall prevalence of internet addiction of 34.53% [24].

These differences could be attributed to the use of different cut-off point scores used to define internet addiction, as well as the easy accessibility of the internet in Sudan. Additionally, the unique circumstances encountered by medical students in Sudan, such as multiple university closures, may drive them to use the internet as a coping mechanism. Moreover, a recent study conducted in Egypt among medical students showed an addiction rate of 68.3%; however, this study used a different measuring tool [8].

While previous studies have often reported a higher prevalence of internet addiction among males, our study revealed a 1.9-fold higher risk in female medical students [11,20,25]. This finding aligns with the survey conducted by Haroon et al. in Pakistan [26] and the study conducted in Qassim, Saudi Arabia [12]. An explanation for this variation could be the cultural contexts of female medical students in Sudan. These contexts could lead to less participation in outdoor activities and a stronger preference for indoor pursuits, which may result in social isolation. Such isolation might drive them towards online platforms for social interaction and emotional support. This hypothesis is supported by a meta-analysis by Mozafar Saadati et al., which found a strong association between social isolation and internet addiction, as individuals often seek to fulfill their emotional needs through online connections [27]. Additionally, the limited availability of recreational activities and alternative stress relief options in their immediate environment could play a significant role.

Interestingly, our study revealed a significantly lower risk of internet addiction among medical students in the fourth and fifth years. This finding could be attributed to the increased engagement in practical activities and clinical rounds at these levels, potentially reducing their susceptibility to excessive internet use. However, other studies did not differentiate between medical students based on their academic years [10,28].

In our study, we examined the level of mental distress and its correlation with internet addiction. We observed a direct positive correlation between internet addiction and psychological distress. This finding is consistent with a study conducted in Iraq, which reported a significant association between excessive internet use and both depression and anxiety [18]. Saikia et al.'s study further provided evidence that individuals addicted to the internet had a 14-fold higher risk of developing depression, were 12 times more likely to experience stress, and were 3.3 times more prone to anxiety compared to those who were not addicted [19]. Additionally, research conducted by Ansari et al. revealed a strong relationship between the level of internet addiction among medical students and psychological distress [13]. Sudanese medical students have faced unique mental health distress due to political instabilities and severe financial deterioration. There is evidence indicating that the prevalence of depression among medical students in Sudan is higher compared to other developing countries [29].

Using logistic regression, we found a 6.4-fold increased risk of internet addiction among medical students who experienced psychological distress. This aligns with several previous studies. For example, Zenebe et al.'s study in Ethiopia noted that undergraduate students experiencing mental distress had a 2.7-fold higher risk of developing Internet Addiction [21]. The study conducted by Anand et al. suggests that psychological distress is a significant risk factor for internet addiction [30]. Similarly, Seo et al. found that people with social anxiety are at an increased risk of experiencing internet addiction [1].

There is a perspective that individuals with depression might turn to the internet as a means to alleviate life dissatisfaction, as engaging in online activities can potentially help reduce negative feelings. This is particularly relevant for medical students, who often face long study hours and high academic stress, leading them to seek relief through online activities. Furthermore, the absence of social support in real life, including friendships and other interpersonal connections, may lead individuals to pursue these needs on the internet, potentially contributing to internet addiction. Consequently, with their limited opportunities for socializing and participating in physical or recreational activities, the internet becomes more attractive for connection and relaxation.

Despite our efforts, this study has some limitations that could be addressed in future research. Firstly, the questionnaire relies on self-reported data, which can be subject to self-reporting bias and may not accurately reflect the actual prevalence of internet addiction among individuals. Additionally, our study's sample was confined to medical students from one university, predominantly female, which may limit the generalizability of our results. However, this study is the first in Sudan to examine internet addiction among medical students from all academic levels. The study utilized a robust sampling technique and provided valuable insights into the prevalence of internet addiction, including its different severities and associated factors among undergraduate medical students, particularly in Sudan.

## Conclusions

The current study revealed a high prevalence of internet addiction among medical students at the National University, which is strongly associated with mental distress. Effective awareness programs, potentially initiated by university authorities, are required to educate medical students about the importance of restricting excessive internet usage and addressing the underlying risk factors. Additionally, studies involving multi-university samples are suggested to obtain more generalizable results, and longitudinal studies to examine the relationship between internet addiction and mental distress more comprehensively and robustly.
